# Haloperidol dose combined with dexamethasone for PONV prophylaxis in high-risk patients undergoing gynecological laparoscopic surgery: a prospective, randomized, double-blind, dose-response and placebo-controlled study

**DOI:** 10.1186/s12871-015-0081-1

**Published:** 2015-07-08

**Authors:** Jin Joo, Yong Gyu Park, Jungwon Baek, Young Eun Moon

**Affiliations:** 1 Department of Anesthesiology and Pain Medicine, Seoul St. Mary’s Hospital, College of Medicine, The Catholic University of Korea, 222 Banpo-daero, Seocho-gu, Seoul, 137-701 Republic of Korea; 2 Department of Biostatistics, College of Medicine, The Catholic University of Korea, Seoul, Republic of Korea

**Keywords:** Combination drug therapy, Haloperidol, PONV, Sedation

## Abstract

**Background:**

Low-dose haloperidol is known to be effective for the prevention of postoperative nausea and vomiting (PONV). However, precise dose-response studies have not been completed, especially in patients at high risk for PONV who require combination therapy. This study sought to identify which dose of haloperidol 1mg or 2mg could be combined with dexamethasone without adverse effects in high-risk patients undergoing gynecological laparoscopic surgery.

**Methods:**

Female adults (*n* = 150) with three established PONV risk factors based on Apfel’s score were randomized into one of three study groups. At the end of anesthesia, groups H0, H1, and H2 were given intravenous (IV) saline, haloperidol 1 mg, and haloperidol 2 mg, respectively. All patients were given dexamethasone 5 mg during the induction of anesthesia. The overall early (0–2 h) and late (2–24 h) incidences of nausea, vomiting, rescue anti-emetic administration, pain, and adverse effects (cardiac arrhythmias and extrapyramidal effects) were assessed postoperatively. The sedation score was recorded in the postanesthesia care unit (PACU).

**Results:**

The total incidence of PONV over 24 h was significantly lower in groups H1 (29 %) and H2 (24 %) than in group H0 (54 %; *P* = 0.003), but there was no significant difference between groups H1 and H2. In the PACU, group H2 had a higher sedation score than groups H1 and H0 (*P* < 0.001).

**Conclusions:**

For high-risk PONV patients undergoing gynecological laparoscopic surgery, when used with dexamethasone, 1-mg haloperidol was equally effective as 2 mg in terms of preventing PONV with the less sedative effect.

**Trial Registration:**

ClinicalTrials.gov (NCT01639599).

## Background

Postoperative nausea and vomiting (PONV) is a common complication of general anesthesia. In high-risk populations, such as females undergoing laparoscopic surgery with opioid patient-controlled analgesia (PCA) for pain control, the incidence can be as high as 79 % [1]. For these high-risk patients, combination therapy using two or more medications of different classes is more effective in preventing PONV than the use of a single anti-emetic [2].

The most widely used combination in current clinical practice consists of a 5-HT_3_ receptor antagonist and dexamethasone [2]. Another choice is the combination of dexamethasone and a butyrophenone, such as droperidol, and this combination is known to be more cost-effective [3]. However, the production of droperidol was banned in several countries after the United States Food and Drug Administration (FDA) issued a black-box warning due to QTc prolongation [4]. The exit of droperidol from the market prompted a search for a replacement, and many reports have considered haloperidol, another butyrophenone, as a substitute and the commonly used doses were 1 or 2 mg IV [3, 5–9]. However, few studies have examined the dose-response of haloperidol [10], especially in combination therapy for high-risk patients. Therefore, we conducted a prospective, randomized, double-blinded study to identify the appropriate dose of haloperidol (1mg vs. 2mg) to use in combination with dexamethasone for preventing PONV in high-risk patients undergoing gynecological laparoscopic surgery.

## Methods

This study was approved by our local ethics committee (Institutional Review Board of Catholic University Seoul Saint Mary’s Hospital, Seoul, Korea, Ref. KC11MISI0335) and registered at ClinicalTrials.gov (Ref: NCT01639599). Written informed consent was obtained from all study subjects. The study subjects were American Society of Anesthesiologists (ASA) physical status I or II females, age 20–65 years, scheduled for gynecologic laparoscopic surgery and intravenous (IV) PCA for postoperative pain control. Based on Apfel’s simplified risk score, the patients in this study had the following three standard PONV risk factors: female, non-smoker, and use of opioid analgesics after surgery. Exclusion criteria were as follows: known allergy or intolerance to the study drug; history of cardiac arrhythmia; psychiatric illness; chronic treatment with a dopamine antagonist; use of opioids or steroids within 1 week of surgery; use of anti-emetic in the 24 h before the study; unable to use the PCA device; gastrointestinal, renal, or hepatic disease; and insulin-dependent diabetes or obesity with a body mass index >35 kg/m^2^.

Using computer-generated codes placed in sequentially numbered, opaque envelopes, the enrolled patients were assigned randomly to one of three groups: group H0 = saline, group H1 = haloperidol 1 mg, and group H2 = haloperidol 2 mg. A nurse anesthetist not involved in the treatment opened the envelopes and prepared the study drugs in identical syringes with a total volume of 2 mL (diluted with saline). The patients and attending anesthesiologists were blinded to the group assignments. Staff who remained blind to the group assignment made the postoperative evaluations.

General anesthesia was induced with 0.5–1 μg/kg remifentanil and 1–2 mg/kg propofol. Orotracheal intubation was performed after administering 0.8 mg/kg rocuronium. All patients received 5-mg dexamethasone IV during induction. Anesthesia was maintained with 0.05–0.15 μg/kg/min remifentanil and 1.2-2 % sevoflurane (endotidal concentration) in 50 % air/oxygen to keep the bispectral index (BIS) value at 40–60. Ventilation was controlled mechanically and adjusted to maintain end-tidal CO_2_ values of 30–40 mmHg. Additional rocuronium was administered as required. The laparoscopy was performed under video guidance with three punctures in the abdomen. Approximately 30 min before the end of anesthesia, the study drug was administered. Residual neuromuscular blockade was antagonized with 10-mg pyridostigmine and 0.4-mg glycopyrrolate IV. The trachea was extubated when the patient was awake. No other opioid was administered during the operation. On arriving at the postanesthesia care unit (PACU), all patients were given acetaminophen 1 g IV for postoperative pain control. The patients were administered IV PCA when discharged from the PACU after 2 h. The PCA regimen consisted of 1-mg fentanyl and 120-mg ketorolac (total volume including saline 100 mL) and was programmed to deliver 1 mL/h as a background infusion and 1 mL per demand, with a 10-min lockout.

Two postoperative time periods were evaluated: 0–2 and 2–24 h after surgery. For the first 2 h, a trained investigator without knowledge of the study group assignment measured the following variables in the PACU: incidence of nausea and vomiting (or retching), pain intensity, rescue anti-emetics, rescue analgesics and incidence of adverse effects such as cardiac arrhythmias or neurological side effects. Nausea was defined subjectively unpleasant sensation associated with awareness of the urge to vomit. Nausea was also graded as tolerable or intolerable. Rescue medication (4-mg ondansetron) was administered to any patient who experienced intolerable nausea or vomiting, or who requested rescue anti-emetics. The primary outcome was the incidence of nausea or vomiting during the study period.

Postoperative pain intensity was measured using a 10-cm visual analogue scale (VAS; 0 = no pain and 10 = the worst pain imaginable). When a patient complained of more pain and requested analgesia, 30 mg ketorolac IV was given (a maximum of 120 mg/day). After discharge from the PACU, data were collected by a blinded investigator every 6 h.

The standard lead ECG was monitored continuously at a paper speed of 25 mm/s and an amplification of 0.1 mV/mm. Heart rate was calculated from three RR intervals preceding the measured QT intervals. The QT intervals were measured manually from the onset of the QRS complexes to the end of the T wave and corrected for the patient’s heart rate using Bazett’s formula $\mathrm{Q}\mathrm{T}\mathrm{c} = \mathrm{Q}\mathrm{T}/\sqrt{\mathrm{RR}}$. The QTc interval was measured pre-operatively and 10 min after each patient arrived in the PACU.

The level of sedation was evaluated immediately, 30, 60, 90, and 120 min after arriving in the PACU. Each patient was asked to assess her level of sedation using a 10-cm VAS (0 = wide awake and 10 = maximally asleep) [11, 12].

### Statistical analysis

The primary endpoint of this study was the incidence of PONV during the study period. In a preliminary study of 15 patients who received dexamethasone and had the same inclusion and exclusion criteria, 60 % of the patients suffered from nausea and vomiting for up to 24 h after gynecological laparoscopic surgery. To obtain an 80 % chance of identifying a 30 % reduction of PONV incidence during the first 24 h after surgery at the level α = 0.05 (two-sided), 43 patients were required for each group. Considering potential dropouts, 50 patients were included in each group. One-way analysis of variance (ANOVA) was used to examine differences in the parametric data among the three groups. If a significant difference was found, the Bonferroni test was used to detect the inter-group differences. Changes in the sedation VAS scores over time were analyzed using repeated-measures ANOVA with a *post hoc* test. Categorical data were analyzed using the chi-square test. The Bonferroni correction was used for all multiple comparisons. *P* < 0.05 was considered statistically significant. SPSS ver. 15.0 for Windows (SPSS, Chicago, IL, USA) was used for the analyses.

## Results

**Fig. 1:**
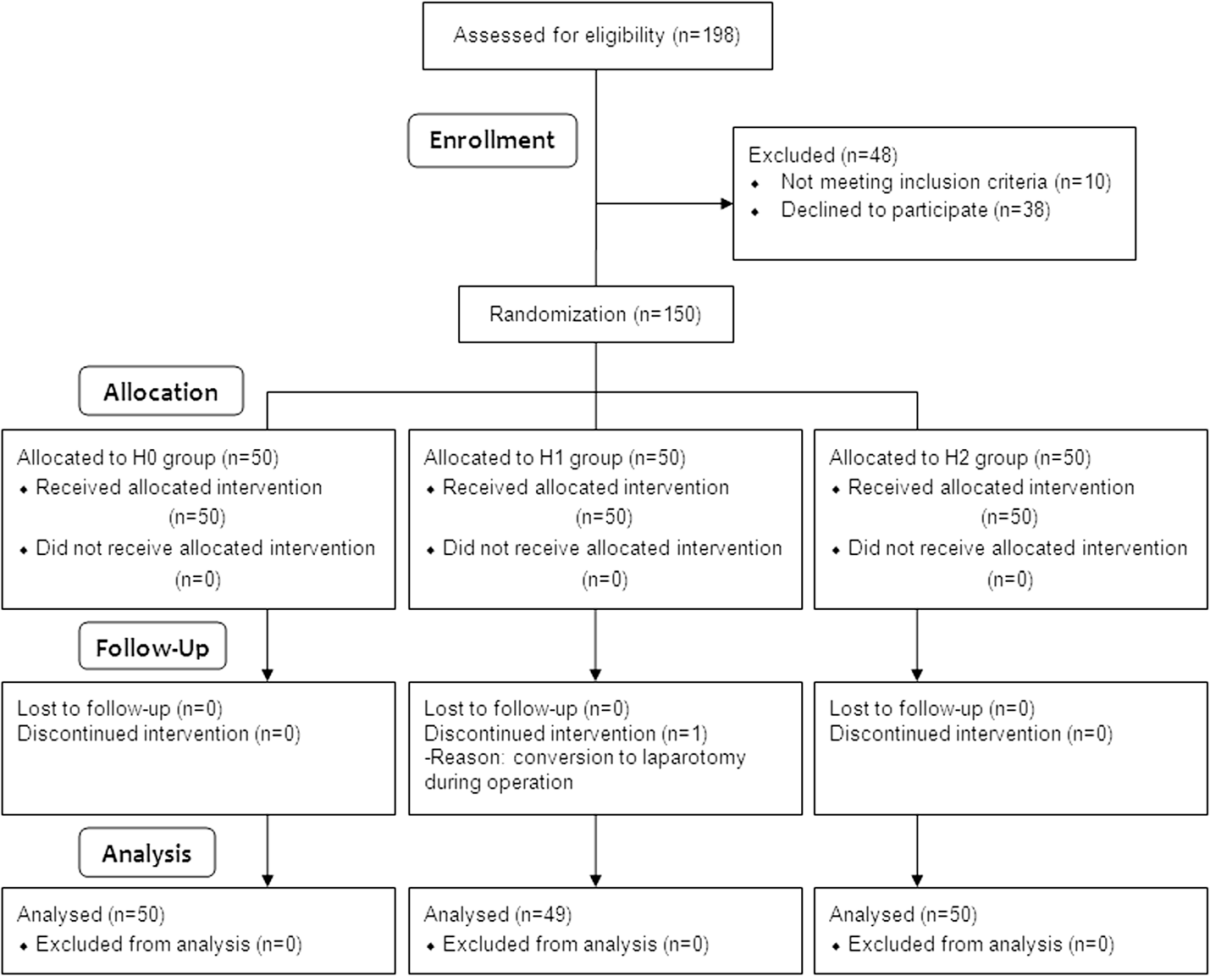
CONSORT diagram showing the flow of participants

Of the 150 patients registered for the study, one was excluded due to an intra-operative conversion to laparotomy (Fig. 1). The remaining 149 patients, consisting of 50, 49, and 50 patients in groups H0, H1, and H2, respectively, completed the study. The patient characteristics, PONV risk factors, type of surgery, duration of anesthesia, intra-operative remifentanil use, 24-h postoperative PCA fentanyl use, postoperative pain severity, and rescue analgesic requirements were similar among the three groups (Table 1).

**Table 1:** Patient characteristics and variables associated with PONV

	Group H0 (*n* = 50)	Group H1 (*n* = 49)	Group H2 (*n* = 50)
Age (year)	40 (20–60)	39 (20–60)	41 (21–58)
Weight (kg)	56.9 (5.7)	55.0 (7.9)	54.4 (5.3)
Height (cm)	158.7 (4.9)	159.1 (5.5)	157.3 (5.5)
ASA I/II	40/10	41/8	39/11
PONV or motion sickness history	26 (52 %)	28 (57 %)	28 (56 %)
Anesthesia duration (min)	150.3 (43.1)	157.9 (39.4)	156.4 (31.4)
Remifentanil consumption (μg)	104.7 (36.2)	97.2 (31.3)	92.5 (29.2)
Surgical type			
Lap. ovarian cystectomy	17 (34 %)	12 (24 %)	13 (26 %)
Lap. hysterectomy	23 (46 %)	22 (45 %)	26 (52 %)
Diagnostic laparoscopy	5 (10 %)	11 (22 %)	6 (12 %)
Lap. myomectomy	4 (8 %)	3 (6 %)	4 (8 %)
Lap. adhesiolysis	1 (2 %)	1 (2 %)	1 (2 %)
PCA fentanyl consumption for 24 h (μg)	470.1 (105.5)	465.6 (102.6)	467.8 (99.5)
Postoperative pain			
0-2 h	4.9 (1.7)	4.8 (1.8)	4.6 (2.1)
2-24 h	2.2 (2.1)	2.8 (1.6)	2.2 (1.9)
Rescue analgesic requirements	15 (30 %)	11 (22 %)	10 (20 %)

Values are means (SD) or number (proportion). No statistically significant difference was observed among the groups

Group H0 = saline, H1 = haloperidol 1 mg, and H2 = haloperidol 2 mg

*PONV* postoperative nausea and vomiting; *Lap* laparoscopic; *PCA* patient-controlled analgesia

Overall, the frequency of PONV within 24 h postoperatively was lower in groups H1 and H2 than in group H0, while there was no statistical difference between groups H1 and H2 (29, 24, and 54 % in groups H1, H2, and H0, respectively; *P* = 0.003). Table 2 summarizes the results at each postoperative time point.

**Table 2:** PONV outcomes

	Group H0	Group H1	Group H2	
	(*n* = 50)	(*n* = 49)	(*n* = 50)	P*
Early time (0–2 h)				
Nausea	15 (30 %)	5 (10 %)†	9 (18 %)	0.04
Vomiting	7 (14 %)	3 (6 %)	4 (8 %)	0.37
Total PONV	17 (34 %)	5 (10 %)†	10 (20 %)	0.02
Rescue anti-emetics	12 (24 %)	3 (6 %)†	6 (12 %)	0.03
Late time (2–24 h)				
Nausea	17 (34 %)	10 (20 %)	9 (18 %)	0.13
Vomiting	6 (12 %)	5 (10 %)	3 (6 %)	0.57
Total PONV	21 (42 %)	11 (22 %)	10 (20 %)	0.03
Rescue anti-emetics	11 (22 %)	7 (14 %)	7 (14 %)	0.48

Values are numbers (proportion). Groups H0 = saline, H1 = haloperidol 1 mg, and H2 = haloperidol 2 mg

*PONV* postoperative nausea and vomiting

*Unadjusted *P* value

†*P* < 0.017, compared with group H0

In the PACU (0–2 h), group H1 had a lower incidence of PONV and reduced requirements for rescue anti-emetics than group H0, while group H2 did not differ significantly from group H0. In the ward (2–24 h), the incidence of PONV was lower in groups H1 and H2 than in group H0, but there was no statistically significant difference between groups H1 and H2 (22 % in group H1 and 20 % in group H2 *vs*. 42 % in group H0).

**Fig. 2:**
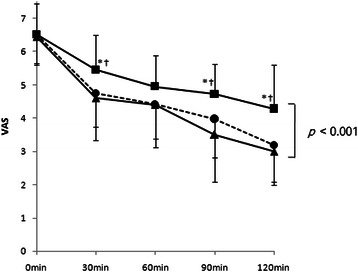
Postanesthesia care unit-sedation scores recorded using a 10 cm visual analogue scale (VAS; 0 = wide awake and 10 = maximally asleep) in patients receiving saline (▲), haloperidol 1 mg (●) or haloperidol 2mg (■). *P < 0.05 compared with saline. †*P* < 0.05 compared with haloperidol 1 mg

The change in the sedation score in the PACU clearly differed among the groups according to the dose of haloperidol. Group H2 had an overall higher sedation score than groups H1 and H0 over the 2-h observation period (*P* < 0.001, Fig. 2), while there was no significant difference between groups H1 and H0. In addition, three patients in group H2 required treatment with ephedrine in the PACU, due to hypotension (systolic 75–80/diastolic 50–55 mmHg).

The QTc intervals before administering the study drug were similar among the groups (group H0, 423.85 ± 21.94 ms; group H1, 425.23 ± 0.21 ms; and group H2, 426.35 ± 15.13 ms). The QTc interval measured in the PACU also did not differ among the groups (group H0, 426.75 ± 19.64 ms; group H1, 430.67 ± 21.92 ms; and group H2, 431.95 ± 23.14 ms). No cardiac arrhythmias were observed in the PACU. No patient showed evidence of twitching, dystonia, akathisia, or other extrapyramidal reactions during the 24-h postoperative observation period.

## Discussion

In this randomized, double-blind study, both 1- and 2-mg haloperidol, in combination with dexamethasone, had PONV-preventing effects during the first 24 h postoperative, compared with dexamethasone monotherapy. However, 2-mg haloperidol resulted in more sedation of patients in the PACU than the 1-mg dose.

According to Apfel’s risk score, each of the following risk factors increases the risk of PONV by 20 %: female, nonsmoker, postoperative opioid use, and a history of PONV or motion sickness [1]. All patients enrolled in our study had an Apfel’s risk score ≥3, so the anticipated incidence of PONV exceeded 60 %. Additionally, pelvic surgery is known to increase risk for PONV. According to the consensus guidelines for managing PONV, these high-risk patients require combination therapy using drugs from two or three different classes [13]. The combination used most commonly is dexamethasone plus a 5-HT_3_ antagonist, while the combination of dexamethasone and a butyrophenone compound is known to be more cost-effective.

Many studies have investigated haloperidol, another butyrophenone. In a meta-analysis of 23 randomized trials, the dosage of haloperidol suggested to be effective at preventing PONV was 0.5–4 mg [14]. However, the delivery methods in this analysis included intravenous (IV) and intramuscular (IM) routes, and this lack of uniformity meant that a precise dose-response relationship could not be identified. Furthermore, although many recent clinical trials have reported on the anti-emetic effect of haloperidol and that the commonly used doses were either 1 or 2 mg IV [3, 5–9], there have been few dose-response studies within this dose range. In our study, we chose 1 mg as the minimum dose based on Dagtekin’s research [15]. They reported that 10 μg/kg haloperidol had a limited effect in female subjects compared with the placebo. The dose range of haloperidol used in that study was ~550-830 μg considering the participants’ weights. Thus, given that a dose less than 1 mg was ineffective in female patients, we used 1 mg as the minimum dose in our study.

Parlow et al. compared the anti-emetic effect of 1- or 2-mg haloperidol IM [10]. However, their study design differed from ours in that they enrolled non-high-risk patients undergoing spinal anesthesia. By contrast, we sought to determine the appropriate dosage of haloperidol in combination with dexamethasone, not causing side effects, for high-risk patients requiring combination therapy after general anesthesia.

In our study, 1- and 2-mg haloperidol with dexamethasone more effectively reduced PONV compared with dexamethasone alone, while no significant differences were detected between the two groups. Interestingly, the incidence of PONV in the PACU was slightly higher in group H2 than in group H1 (20 % *vs*. 10 %: not statistically significant).

In general, various factors can affect postoperative sedation, including the type and duration of anesthesia, patient age, and the use of narcotics in the PACU. In our study, short-acting sevoflurane and remifentanil were used, and the depth of anesthesia was maintained at BIS 40–60. Pain control in the PACU was achieved with non-narcotic analgesics, and opioid PCA was applied at the time of discharge from the PACU. The duration of anesthesia and age distribution of the patients were similar among the three study groups. These facts suggest that the observed difference in sedation was probably attributable to the dosage of haloperidol. In our study, the sedation scores of patients in group H2 were significantly higher than those in group H1, as well as in the control group, while there was no difference between group H1 and the control group. The differences in sedation scores became more apparent 90 and 120 min after arriving in the PACU, when the effect of the inhaled anesthetics had ended. These results do not agree with a previous study in which 2-mg haloperidol did not have a sedative effect, compared with normal saline [6]. In that study, however, the level of sedation was evaluated only once (30 min after surgery), and no specific data on sedation were presented.

This delayed sedative effect is likely related to the long plasma half-life of haloperidol, compared with droperidol (18 h for haloperidol vs. 2 h for droperidol) [16, 17]. Therefore, it has been suggested that the drug has a longer duration of action, even when administered at low doses [18]. Forsman reported that the sedative effects reached a maximum during the first 1-h distribution period after IV administration [19]. This sedative effect would increase in the PACU when combined with residual anesthetics following general anesthesia. Therefore, the dose of haloperidol in patients receiving general anesthesia should be considered carefully.

Buttner et al. reported that only 1 of 806 patients who received 0.25–5-mg haloperidol IV experienced extrapyramidal symptoms with a 4-mg dose [14]. In our study, no patients showed neurological side effects during the postoperative 24 h. In the same report, 1397 patients received various haloperidol regimens, but no cardiac arrhythmia was reported [14]. In our study, the QTc interval was not significantly different among the groups after administering the study medication, and there was no evidence of any adverse effect on cardiac rhythm during the first postoperative 2 h. However, our study design cannot lead one to the conclusion that low-dose haloperidol is entirely safe. A large number of subjects are necessary to exclude all possible adverse effects and so this drug should still be used with caution in critically ill patients.

There were a number of limitations to our study. First, there was no complete control group. All patients, including the control group, were administered 5-mg dexamethasone IV during induction. It was considered unethical to exclude high-risk patients from prophylactic management of PONV. Furthermore, these two anti-emetics have different mechanisms of action. Haloperidol acts by antagonizing D2 receptors in the chemoreceptor trigger zone of the medulla [20], while dexamethasone activates glucocorticoid receptors in the solitary tract nucleus of the medulla [21]. Second, the primary endpoint of this study was the detection of differences in anti-emetic effect, but not sedative effect, according to the haloperidol dosage. Consequently, to measure the sedated state, a simple VAS score was used instead of more sophisticated methods that might be able to detect more subtle effects of the drug dosage. Nevertheless, our results are justified since a VAS has been proven valid for measuring sedation change over time and has been used in many studies [11, 12]. Third, our study had a relatively small sample size compared to a complete dose-response study. Thus, our results must be considered as exploratory results, rather than confirmatory results. In order to generalize our results, a full dose-response study may be necessary.

## Conclusions

For high-risk patients requiring combination anti-emetic therapy after general anesthesia, when used with dexamethasone, 1-mg haloperidol was more appropriate in terms of preventing PONV with less sedative effect than 2-mg haloperidol. Importantly, increasing the dose beyond 1 mg did not have additional positive effects, but might induce negative side effects. However, in order to confirm our exploratory results, a well-designed, large-sample, dose-response study is necessary in future.

## Abbreviations

PONV: Postoperative nausea and vomiting
PACU: Postanesthesia care unit
IV: Intravenous
PCA: Patient-controlled analgesia
ECG: Electrocardiogram
ANOVA: One-way analysis of variance (ANOVA)
IM: Intramuscular
